# Pylorus-preserving total pancreatectomy for metastatic renal cell carcinoma: a case report

**DOI:** 10.1186/s13256-015-0654-0

**Published:** 2015-09-16

**Authors:** Hiroaki Kitade, Hidesuke Yanagida, Masanori Yamada, Takashi Matsuura, Kazuhiko Yoshioka, Sohei Satoi, Yoichi Matsui, Masanori Kon

**Affiliations:** 1 Department of Surgery, Kansai Medical University, Takii Hospital, 10-15 Fumizono-cho, Moriguchi, Osaka 570-8507 Japan; 2 Department of Surgery, Kansai Medical University, 2-5-1 Shin-machi, Hirakata, Osaka 573-1191 Japan

**Keywords:** Metastasis, Pancreas, Pylorus-preserving total pancreatectomy, Renal cell carcinoma

## Abstract

**Introduction:**

Resectable isolated multiple metastases to the pancreas from renal cell carcinoma are rare. In this report, we describe a patient with multiple metastases of renal cell carcinoma to the pancreas who was treated with pylorus-preserving total pancreatectomy.

**Case presentation:**

The patient was a 58-year-old Asian woman who had undergone right nephrectomy for renal cell carcinoma 20 years earlier. In 2008, she was diagnosed with multiple metastases of renal cell carcinoma to the pancreas by abdominal computed tomography during routine follow-up for renal cell carcinoma. ^18^F-2-fluoro-2-deoxyglucose positron emission tomography/computed tomography showed no accumulation in her body other than the pancreas. Because of concerns about her quality of life after total pancreatectomy, she underwent pylorus-preserving total pancreatectomy. After the resection, her control of blood sugar and quality of life were generally satisfactory. She died as a result of gastrointestinal bleeding 35 months after undergoing pancreatectomy.

**Conclusions:**

Pancreatic metastasectomy should be considered, even for multiple metastases, when the primary tumor is renal cell carcinoma and the metastatic lesions are isolated.

## Introduction

Metastatic tumors of the pancreas are rare. Most patients with metastases to the pancreas are not candidates for resection, because the lesions are often widespread. It has been reported that only 1.8 % of patients who undergo pancreatectomy do so for metastatic cancer [1]. The most common cancers reported to metastasize to the pancreas include renal cell carcinoma (RCC), colon cancer, melanoma, sarcoma, breast cancer, and lung cancer [2, 3], with the kidneys being the most common primary tumor site (70.5 %) [4]. RCCs frequently metastasize only to the pancreas, and these metastases may occur a long time after nephrectomy.

Surgical resection has been reported to improve the prognosis of patients with RCC [5]. Only 11 % of metastatic RCCs to the pancreas have been reported to be multifocal or to have an unsuspected location. Therefore, only 18.6 % of patients who undergo surgery for these metastases undergo total pancreatectomy (TP) [4]. In this case report, we describe a patient who underwent pylorus-preserving total pancreatectomy (PPTP) for multiple metastases to the pancreas from RCC 20 years after nephrectomy.

## Case presentation

**Fig. 1:**
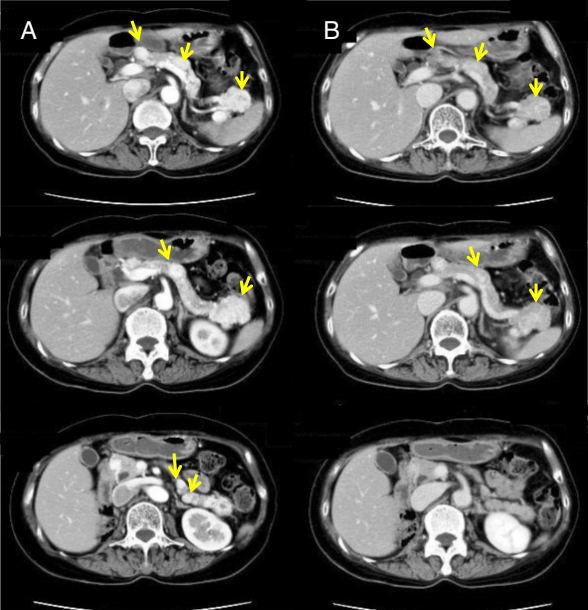
Contrast-enhanced abdominal computed tomography revealed multiple stained nodules in the pancreas (yellow arrows). **a** Arterial phase. **b** Late phase

**Fig. 2:**
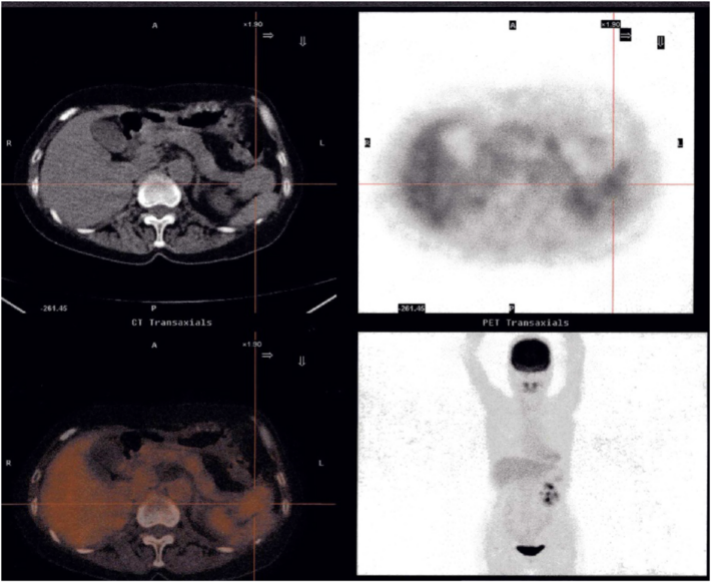
^18^F-2-fluoro-2-deoxyglucose positron emission tomography/computed tomography showing ^18^F-2-fluoro-2-deoxyglucose accumulation in the tail of the pancreas (standardized uptake value, 2.5)

**Fig. 3:**
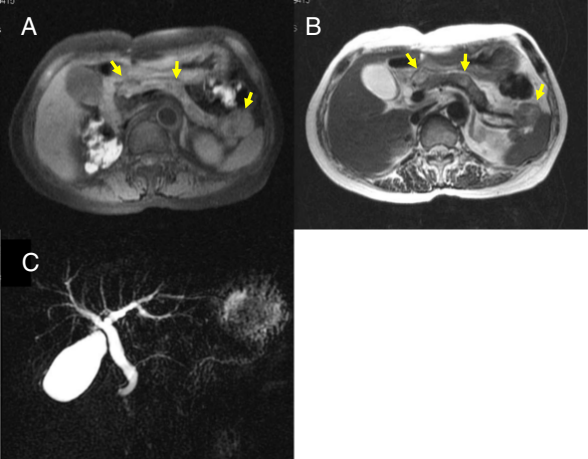
Abdominal magnetic resonance imaging scans (**a** T1 weighted, **b** T2 weighted MRI image) showing multiple nodular legions in the pancreas head, tail, and body (yellow arrows). There was no evidence of dilatation of the main pancreatic duct or bile duct (**c**)

A 58-year-old Asian woman was admitted to our hospital for multiple nodular legions in the pancreas. She had undergone right nephrectomy for RCC 20 years earlier. Since then, she had undergone soft tissue resection of the right shoulder (2005), partial left nephrectomy (2006), and partial chest wall resection (2007) for metastases from RCC, and she was started on interferon therapy in 2007. In 2008, during routine follow-up, abdominal computed tomography (CT) revealed multiple space-occupying legions in the pancreas, but she had no subjective symptoms. Her carcinoembryonic antigen and carbohydrate antigen 19-9 levels were within normal limits. Contrast-enhanced abdominal CT revealed multiple stained nodules in the pancreas (Fig. 1). ^18^F-2-fluoro-2-deoxyglucose positron emission tomography/computed tomography (FDG-PET/CT) showed FDG accumulation in the tail of the pancreas (standardized uptake value, 2.5) (Fig. 2), but no other accumulations of FDG elsewhere in her body. Abdominal magnetic resonance imaging (MRI) showed three stained nodular legions (one each in the head, tail, and body of the pancreas), but no evidence of dilatation of the main pancreatic duct or bile duct (Fig. 3). Preoperative differential diagnoses included pancreatic endocrine tumor and metastatic carcinoma. On the basis of these findings and her previous medical history, she was diagnosed with multiple isolated metastases to the pancreas from RCC.

**Fig. 4:**
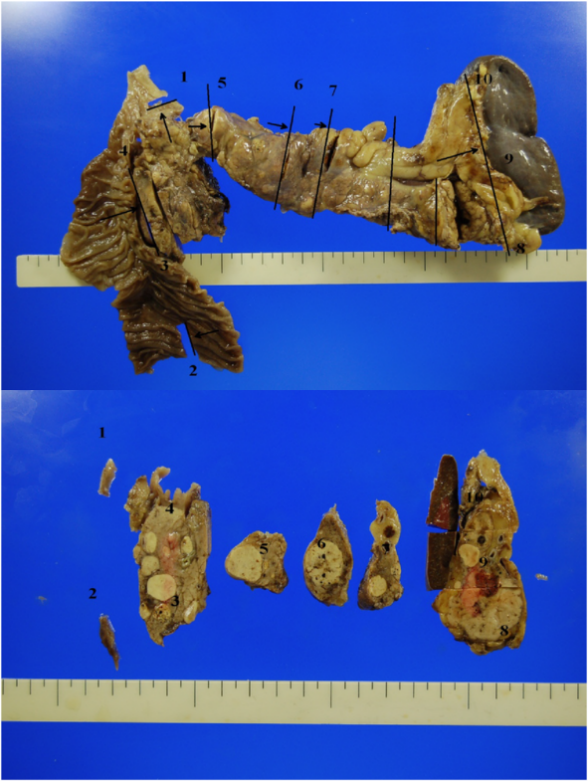
Macroscopic findings. More than ten macroscopic lesions were observed

**Fig. 5:**
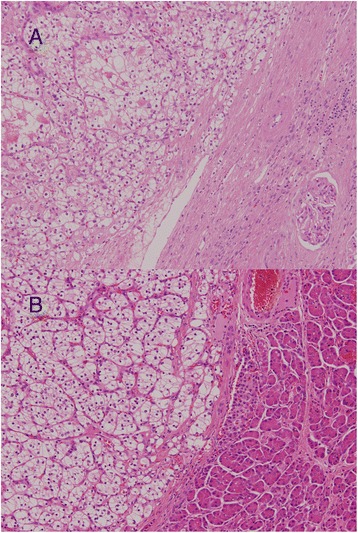
Microscopic findings. The pathological diagnosis was compatible with metastatic clear cell renal cell carcinoma (hematoxylin and eosin stain; original magnification, ×100). **a** Primary renal cell carcinoma resected 20 years earlier. **b** Metastatic renal cell carcinoma

As metastases occurred while the patient was being treated with interferon, surgery was indicated. Intraoperative ultrasonography showed more than four nodules in the pancreas from the head to the tail, but there was no evidence of lymph node swelling or peritoneal dissemination. She underwent PPTP with splenectomy. Because all blood supply to the stomach comes from the left gastric artery via intramural vessels and all blood drains from the stomach through the left gastric vein, close attention was paid to preservation of these vessels. Her pancreas contained ten macroscopic and more than eleven microscopic metastatic lesions (Figs. 4 and 5). Their pathological diagnosis was compatible with metastatic clear cell RCC, similar to the primary RCC resected 20 years earlier (Fig. 5).

Two weeks after PPTP, the patient fell during a hypoglycemic episode and broke her right femur. Subsequently, however, her control of blood sugar was generally satisfactory. Adjuvant therapy consisted of interleukin (IL)-2 (70,000 U/week) for 2 years, after which IL-2 therapy was discontinued because the patient was experiencing severe nausea and vomiting. There was no evidence of RCC recurrence 28 months after PPTP, but she changed hospitals thereafter. We were informed by a local hospital that she died as a result of gastrointestinal bleeding 35 months after pancreatic resection.

## Discussion

Resectable isolated multiple metastases to the pancreas from RCC are rare. Most of these patients are not candidates for surgical treatment. In this report, we describe a rare case of isolated multiple metastases to the pancreas from RCC treated by PPTP.

Diagnosis of metastases to the pancreas from RCC is often difficult, and thus knowledge of a patient’s medical history is important when the pancreatic mass is initially detected. In our patient, however, the diagnosis was relatively easy because she had been followed for a long period of time after nephrectomy. In general, contrast-enhanced CT, MRI, and FDG-PET/CT are used for the differential diagnosis of nodules in the pancreas. Metastases to the pancreas from RCC are detected as hypervascular tumors on contrast-enhanced CT and dynamic MRI and as accumulations of FDG. Small-sized metastatic RCCs, less than 15mm to 20mm in diameter, have been reported to be homogeneously enhanced [6]. In our patient, small-sized metastatic RCCs were not detected preoperatively by contrast-enhanced CT or MRI. FDG-PET/CT is useful for determining the need for surgery because it can exclude distant metastases. However, accumulation of FDG is low, with the number of actual tumors in the resected specimen generally greater than the number determined by FDG-PET/CT. For example, FDG-PET in our patient showed metastatic lesions only in the tail of the pancreas, suggesting that FDG-PET may not be suitable for the detection of metastatic RCCs in the pancreas. Endoscopic ultrasound-guided fine-needle aspiration biopsy is also used for the definitive diagnosis of metastases of RCC. This was deemed unnecessary in our patient because of her history of RCC, the presence of hypervascular tumors on contrast-enhanced CT, and the local accumulation of FDG, all of which suggested multiple isolated metastases to the pancreas from RCC.

The kidney is the most common primary tumor site (70.5 %) of metastases to the pancreas [4], with many RCCs metastasizing only to the pancreas and many metastases occurring a long time after nephrectomy. The pancreatic metastases in our patient occurred 20 years after initial resection for RCC. Surgical resection has been reported to improve the prognosis of patients with RCC [5]. The 5-year overall survival rate after pancreatectomy for RCC metastases has been reported to be 42 %, much higher than after pancreatectomy for metastases of other cancers. Medical therapies for metastases to the pancreas from RCC include interferon, chemotherapy, and sunitinib, although surgical resection has been found to be superior.

The surgical procedure to choose depends on the location of the metastases. Distal pancreatectomy is the treatment of choice for patients with solitary lesions in the body or tail of the pancreas, whereas pancreatoduodenectomy is usually performed in patients with solitary lesions in the head of the pancreas. In general, TP is performed in patients with widespread or multiple tumors because it is as safe as the pylorus-preserving Whipple procedure for the treatment of benign and malignant neoplasms of the pancreas [7]. TP with or without pylorus preservation has been reported to be safe in patients with metastatic RCC, and this procedure, along with adequate medical support and appropriate education after TP, should result in good control of endocrine and exocrine pancreatic insufficiency as well as a good quality of life [8]. There are several options for TP, with or without splenectomy and with or without pylorus preservation. PPTP is a standard organ-preserving procedure for neoplasms of the entire pancreas. We elected to perform PPTP rather than standard TP plus splenectomy in this patient, for several reasons. First, lymph node dissection was not necessary, owing to the hematogenous metastases of RCC [9]. Second, hypoglycemia was found to be lower after PPTP than after TP [10]. Finally, the incidence of late complications, including uncontrollable diabetes, diarrhea, and malnutrition, was reported to be lower after PPTP than after TP [10]. TP causes loss of endocrine and exocrine functions. Authors who compared standard TP with PPTP for pancreatic cancer found no differences in the rates of early complications, including delayed gastric emptying and cholangitis [10]. Although the rate of late complications was higher in patients who underwent standard TP (9 of 13) than in those who had PPTP (3 of 10), the difference was not statistically significant. However, serum albumin level and percentage of preillness body weight 6 months after resection were significantly higher in patients who underwent PPTP. Several previous case reports have described the use of PPTP for isolated metastases to the pancreas from RCC [11, 12].

Another organ-preserving TP procedure for pancreatic neoplasms is duodenum-preserving total pancreatectomy (DPTP), which was performed on a patient with multiple metastases of the pancreas from RCC [13]. Preservation of the arterial arcade of the posterior pancreas is necessary for the blood supply of the duodenum and common bile duct, but this procedure is difficult to perform in some patients. It is also unclear whether preservation of the duodenum results in good control of blood sugar and good quality of life. The difficulties involved in performing DPTP, coupled with its as yet undetermined benefits, suggest that PPTP should be performed in patients with isolated multiple metastases to the pancreas from RCC. However, additional studies comparing these two procedures for this indication are necessary.

## Conclusions

In this case report, we describe the benefits of surgical resection in a patient with multiple isolated pancreatic metastases from RCC. Organ-preserving TP is the treatment of choice for these patients, as shown by their better quality of life after resection.

## Consent

Written informed consent was obtained from the patient for publication of this case report and accompanying images. A copy of the written consent is available for review by the Editor-in-Chief of this journal.

## Abbreviations

CT: Computed tomography
DPTP: Duodenum-preserving total pancreatectomy
FDG-PET/CT: ^18^F-2-fluoro-2-deoxyglucose positron emission tomography/computed tomography
IL-2: Interleukin-2
MRI: Magnetic resonance imaging
PPTP: Pylorus-preserving total pancreatectomy
RCC: Renal cell carcinoma
TP: Total pancreatectomy
